# Effects of treadmill exercise on PI3K/AKT/GSK-3β pathway and tau protein in high-fat diet-fed rats

**DOI:** 10.20463/jenb.2018.0002

**Published:** 2018-03-31

**Authors:** Jae-Hoon Jeong, Eun-Bum Kang

**Affiliations:** 1. Department of Physical Education, Hanyang University, Seoul Republic of Korea; 2. Division of Sports Science, Daejeon University, Daejeon Republic of Korea

**Keywords:** High-fat diet, Treadmill exercise, PI3K/AKT/GSK-3β pathway, tau protein, cognitive function

## Abstract

**[Purpose]:**

This study aimed to clearly evaluate the effects of obesity on cerebral health. Thus, we induced obesity in rats using a long-term high-fat diet (HFD), then investigated its effects on insulin signaling and tau hyperphosphorylation. Additionally, we examined the effects of 8 weeks of treadmill exercise (TE) on insulin signaling and tau hyperphosphorylation.

**[Methods]:**

Rats were separated into Normal Diet-Control, HFD-Control, and HFD-TE groups. TE loads were gradually increased. A passive avoidance test was used to evaluate cognitive function. Western blots were used to examine the abundance of the insulin receptor,phosphoinositide 3-kinase, protein kinase B, glycogen synthase kinase-3β, and tau proteins in the cerebral cortex; immunohistochemical analyses were used to examine the abundance of hyperphosphorylated tau in the cerebral cortex.

**[Results]:**

TE in HFD-fed rats resulted in a significant lowering of bodyweight, abdominal visceral fat (AVF), the area under the glucose response curve, and the homeostatic model assessment-insulin resistance index, while it improved working memory. In addition, TE in HFD-fed rats decreased tau hyperphosphorylation and aggregation, while increasing insulin signaling-related protein activity.

**[Conclusion]:**

After a 20-week HFD, the experimental animals exhibited increased weight, as well as impaired insulin resistance and blood glucose metabolism. HFD rats demonstrated abnormal insulin signaling and tau hyperphosphorylation in the cerebral cortex, as well as memory impairments that suggested reduced cerebral function. However, TE reduced AVF, improved insulin resistance in the peripheral tissues by increasing insulin sensitivity, and alleviated memory impairments by restoring insulin signaling and reducing tau hyperphosphorylation in the cerebral cortex.

## INTRODUCTION

Obesity, which is defined as a state of having excessive fat tissue that is caused by overnutrition, a sedentary lifestyle, and other environmental factors, is an important risk factor for various metabolic diseases^1,2^. Several studies have shown that obesity is closely related to metabolic diseases such as type 2 diabetes, hypertension, and cardiac disease. Recently, obesity has additionally been linked with various neurological diseases including sleep apnea, depression, and anxiety^3, 4^ as well as Alzheimer’s disease (AD) and other cognitive dysfunctions^5^.

The functions of insulin in peripheral tissues are well understood, while the role of insulin in the central nervous system, especially within the brain, is being re-evaluated. In the brain, insulin is important in the production, development, and activation of neurons; thus, it affects memory and learning in humans^6^. Recent findings have suggested that brain insulin dysfunction might be a cause of AD^7,8^. Under normal circumstances, brain insulin affects the development and activation of neurons and improves cognitive function^6^. However, overeating-induced obesity results in impaired brain insulin signaling, which may lead to cognitive dysfunction and ultimately neurodegenerative disease. Obesity has been found to reduce insulin/insulin-like growth factor-1 activation and increase the levels of tumor necrosis factor-α in the brain, which then increases neural inflammation^9-11^. In other words, oxidative stress^12^ and inflammatory responses are enhanced owing to food-induced obesity, and cerebrospinal insulin resistance is triggered through various pathological phenomena originating from these peripheral tissues. Results from previous studies showing that inflammatory responses in brain tissues became stronger after the extended feeding of a high-fat diet (HFD) support the close association of obesity with brain health parameters including memory and cognitive function^13^.

The molecular mechanisms underlying the relationship between brain insulin dysfunction and neurodegenerative disease are not yet clear. However, brain insulin dysfunction is expected to lead to increased amyloid-β production via the mitogen-activated protein kinase pathway as well as tau hyperphosphorylation via the insulin receptor (IR) and phosphoinositide 3-kinase (PI3K)/protein kinase B (Akt)/glycogen synthase kinase-3β (GSK-3β) pathways. Notably, GSK-3β is associated with the production of hyperphosphorylated tau, which is found in paired helical filaments. Hyperphosphorylated tau is located downstream of the Akt signaling pathway, and tau protein exhibits decreased activity following phosphorylation by Akt^14,15^; thus, an association is expected between tau hyperphosphorylation and insulin. Hence, if brain insulin resistance is a cause of AD, then detailed studies of brain insulin signaling might yield information regarding the alleviation of brain insulin resistance, which could lead to improvements in the prevention and treatment of AD.

Recent studies have shown increased amyloid-β deposition, tau hyperphosphorylation, and memory impairments after obesity was induced by an HFD in transgenic mouse models of AD^16-18^. Importantly, these findings suggest that an HFD accelerates AD. However, because these studies used transgenic animal models that regularly display symptoms of dementia, they were not able to evaluate the effects of obesity-induced insulin dysfunction.

Therefore, to clearly evaluate the effects of obesity on cerebral health in the present study, we used a longterm HFD (20 weeks) to induce obesity in rats; then, we investigated the effects of obesity and subsequent treadmill exercise (TE) on brain insulin signaling and tau hyperphosphorylation.

## METHODS

### Experimental animals

This study protocol was approved by the Animal Ethics Review Board of H-University (KNSU-IACUC-2015-08). The experimental animals were 8-week-old (w.o.) male Sprague–Dawley rats (Koatech Co., Ltd., Gyeonggi-do, Korea) that were reared to 24 w.o. in the animal rearing room at H-University (temperature, 22 ± 2°C; humidity, 50 ± 5%; light-dark cycle, 12 h). When the rats (*n* = 24) were24 w.o., they were supplied with water and a highfat feed (D12482; carbohydrates: 20%, fat: 60%, protein: 20%; Central Lab. Animal, Inc., Seoul, Korea) *ad libitum* for 20 weeks to induce obesity. The rats were assigned to one of the following three groups: Normal Diet-Control (ND-Con, *n* = 12), HFD-Control (HFD-Con, *n* = 12), or HFD-TE (*n* = 12).

### Treadmill exercise

The HFD-TE group underwent acclimation for 30 min/day for 4 days on a rodent treadmill (DJ2-242; Daejong Instrument Industry Co., Ltd., Seoul, Korea) that was fixed at an incline of 0%. After acclimation, the rats underwent training for 5 days per week for 8 weeks. The incline was fixed at 0%, and the load was gradually increased (first 5 min: 8 m/min; next 5 min: 11 m/min; last 20 min: 14 m/min). The TE protocol used in this study was identical to the moderate-intensity exercise program presented by Kang et al.^13^.

### Passive avoidance task

A passive avoidance task was performed before and after TE to evaluate cognitive function. The passive avoidance task was conducted in a light chamber (18 × 18 × 25 cm), which was lit by a bright white light, and a dark chamber (18 × 18 × 25 cm), which was completely dark. The floor of the dark chamber was fitted with a steel shock floor, and a 4-cm-diameter circular hole in the wall between the two chambers was covered with a guillotine door. Each rat was placed by itself in a different cage for 1 min before the experiment. The rat was then placed in the light chamber and allowed to acclimate for 10 s before the guillotine door was opened to allow the rat to explore freely. Once the rat had all four feet inside the dark chamber, the door was swiftly closed. The time taken for the rat to enter the dark chamber (latency time) was measured, and an electric shock (0.5 mA) was given for 2 s. After 5 s, the rat was removed and placed back in the rearing cage. After 72 h, the experiment was repeated using the same methods, and the latency time (up to 300 s) was recorded.

### Body weight and oral glucose tolerance test

The bodyweights (BWs) of the rats were measured twice per week throughout the study, using laboratory scales. The oral glucose tolerance test was performed 12 h after the completion of the 8 weeks of TE. When the rats were in a fasted state, a 30% glucose solution (1 g/kg) was administered orally. Thereafter, blood was collected from the tail vein five times (at 0, 30, 60, 90, and 120 min), and blood glucose was measured using a glucose meter. The blood glucose data were used to calculate the total area under the glucose response curve (AUC0–120, mg/dL/min), which was analyzed using the NCSS 2007 program (NCSS, Kaysville, UT, USA).

### Tissue sample collection

Tissue samples were collected 24 h after the completion of the 8 weeks of TE. Anesthesia was induced in seven rats by intraperitoneal injections of a ketamine/xylazine mixture (2:1, 10 mg/kg) and brain tissue, blood, and abdominal tissue were sampled to measure protein expression. Brain tissue samples that were collected from the cerebral cortex were immediately frozen in liquid nitrogen and stored at −80°C until analysis. Blood was collected from the heart; then, serum was isolated by centrifugation for the glucose and insulin analyses. The abdomen was opened and abdominal visceral fat (AVF) samples were collected, washed in cold physiological saline, and dried with gauze before weighing. For immunohistochemical analysis, five rats were anesthetized and the thoracic cavity was opened. Phosphate-buffered saline (PBS; 50 mM) was perfused via the left ventricle for 10 min, which was followed by perfusion fixation with 4% paraformaldehyde in 100 mM PBS. After this perfusion fixation, each rat’s brain was extracted and submerged in 4% paraformaldehyde fixative for 4 h at 4°C. The fixed brain tissue was then submerged in a 30% sucrose solution for 2 days.The anatomical location (from −4.88 to −1.76 mm from the bregma) including the cortex of the fixedcerebral cortex and hippocampus was extracted using Rodent Brain Matrix(RBM-4000C, 1 mm coronal section; ASI Instruments, Inc., Warren, MI, USA)and continuous coronal slices 40 μm in thickness were obtained using a freezing microtome (Leica, Nussloch, Germany).

### Glucose and insulin concentration analyses

Glucose and insulin concentrations were analyzed using a glucose hexokinase kit (Bayer, Pittsburgh, PA, USA) and a rat insulin kit (Mercodia AB, Uppsala, Sweden), respectively, in accordance with the manufacturers’ instructions. The homeostatic model assessment (HOMA)-insulin resistance (IR) index was calculated using the glucose and insulin concentrations, according to the following equation: HOMA-IR = (fasting serum glucose (mmol/L) × fasting serum insulin (μU/mL))/22.5

### Western blotting

The cerebral cortex was isolated from the previously harvested rat brain tissue, homogenized in lysis buffer, and then centrifuged at 13,000 ×g for 15 min at 4°C. The supernatant was collected andthe concentration of total protein was measured using the Bradford method. Protein samples (30 μg) were subjected to electrophoresis in 7% and 10% sodium dodecyl sulfate-polyacrylamide gels before being transferred to polyvinylidene difluoride membranes (GE Healthcare Bio-Sciences, Pittsburgh, PA, USA). Membranes were then blocked for 1 h at room temperature in a Tris-buffered saline with Tween® 20 (TBS-T) solution containing 3% bovine serum albumin and incubated with the relevant primary antibody (Table 1) for at least 12 h at 4°C. The next day, the membrane was washed three times for 10 min each time in TBS-T before incubation with the relevant secondary antibody for 1 h at room temperature; this was followed by three washes for 10 min each in TBS-T. Finally, the western blotting luminol reagent (sc-2048; Santa Cruz Biotechnology, Inc., Dallas, TX, USA) was added to the membrane. After incubation for 1 min to facilitate chemiluminescence, the membrane was scanned using an image analysis system (ChemiDoc™ XRS; Bio-Rad Laboratories, Inc., Hercules, CA, USA); Quantity One 1-D analysis software (Bio-Rad Laboratories, Inc.) was used to calculate the concentration of protein.

**Table 1.** Primary antibody list

Antibody	Source	Vender	Catalog No.
Total-Tau	mouse monoclonal	Santa Cruz	sc-32274
p-TAU (Ser 202, Thr 205)	mouse monoclonal	Thermo Fisher	MN1020
p-Tau (Thr 231)	rabbit polyclonal	Sigma-Aldrich	T7194
p-Tau (Ser 396)	rabbit monoclonal	Abcam	Ab109390
p-Tau (Ser 404)	rabbit monoclonal	Abcam	Ab92676
p-Tau (Ser 199/202)	rabbit polyclonal	Abcam	Ab9674
Insulin Receptor β	rabbit monoclonal	Cell signaling	#3025
p-Insulin Receptor β	rabbit polyclonal	Abcam	ab60946
PIK3	rabbit monoclonal	Cell signaling	#4257
p-PI3K	rabbit polyclonal	Abcam	ab182651
AKT	rabbit polyclonal	Cell signaling	#9272
p-AKT	mouse monoclonal	Cell signaling	#4051
GSK-3β	rabbit monoclonal	Cell signaling	#9315
p-GSK-3β	rabbit monoclonal	Cell signaling	#9323
α-Tubulin	mouse monoclonal	Santa Cruz	sc-5286

### Immunohistochemical analyses

Five animals were used in the immunohistochemical analyses, and eight slices were selected from each group. Sections were analyzed using the free-floating method. After washing the sections three times for 10 min each time in 0.01 M PBS, each slice was incubated for 8 min at 80°C in a beaker containing 0.01 M sodium citrate, followed by blocking for 40 min in 10% normal donkey serum. After blocking, each slice was incubated with the primary antibody (p-Tau, Ser202/Thr205) for 12 h overnight at 4°C, followed by three washes for 5 min each time in 0.01 M PBS. The sections were then incubated with a horseradish peroxidase-conjugated goat anti-mouse antibody for 1 h at room temperature. The samples were washed an additional three times for 5 min each in 0.01 M PBS, then incubated for 30 min in VECTASTAIN®Elite® ABC kit reagent (Vector Laboratories, Inc., Burlingame, CA, USA) at room temperature and washed a further three times for 5 min each time in 0.01 M PBS. Finally, using a DAB peroxidase substrate kit (SK-4100; Vector Laboratories), 3,3ʹ-diaminobenzidine tetrahydrochloride was diluted to 0.02% in 0.05 M Tris buffer (pH 7.6). Next, 0.02% hydrogen peroxide was added and the samples were stained for 5 min. The stained tissue slices were mounted and analyzed using a light microscope (DM-2500, Leica Microsystems, Wetzlar, Germany).

### Data processing

The data in this study are presented as the mean ± standard deviation and were calculated using SPSS for Windows (version 18.0; IBM Corporation, Armonk, NY, USA). Differences between the groups were analyzed using one-way analysis of variance (ANOVA), and significant differences were verified using Bonferroni posthoc testing. The level of statistical significance was set at α=.05.

## RESULTS

### The effects of TE on the BW, AUC, HOMA-IR, and AVF/BW of the HFD-fed rats

The changes in BW that occurred during the HFD and TE periods are shown in Table 2. First, the initial BWs during the HFD period (at 24 w.o.) were not significantly different among the ND-Con, HFD-Con, and HFD-TE groups [*F*_(2,35)_=1.147, *p*= 0.330]. However, the final BWs during the HFD period (at 43 w.o.) exhibited significant differences among the groups [*F*_(2,35)_=19.771, *p*=.001]. The BWs in the first week of TE (at 44 w.o.) also exhibited significant differences among the three groups [*F*_(2,35)_=21.227, *p*=.001]; likewise, significant differences in BWs were observed among the groups after eight weeks of TE (at 51 w.o.) [*F*_(2,35)_=20.956, *p*=.001]. AUC results that were obtained using an oral glucose tolerance test revealed significant differences among the ND-Con, HFD-Con, and HFD-TE groups [*F*_(2,35)_=8.728, *p*=.001]. HOMA-IR results also showed significant differences among the three groups [*F*_(2,20)_=24.271, *p*=.001]. Finally, the AVF/BW ratio also exhibited significant differences among the ND-Con, HFD-Con, and HFD-TE groups [*F*_(2,20)_=80.823, *p*=.001].

**Table 2.** Comparisons of body weight, AUC, HOMA-IR and AVF/body weight

Metabolic parameter		ND-Con	HFD-Con	HFD-TE
During HFD	Initial BW(g)	454.92±18.87	464.00±23.52	469.42±27.85
	Final BW(g)	559.33±35.85	693.08±75.26*	695.08±63.83*, **
During TE	Initial BW(g)	562.08±33.45	698.83±73.49*	699.25±63.90*, **
	Final BW(g)	582.33±34.99	725.42±68.29*	693.92±61.83*, **
AUC		39436.25±7858.09	54021.25±6758.97*	45137.50±7292.88**
HOMA-IR		1.57±0.37	5.62±1.50*	3.99±1.11*, **
AVF/body weight (%)		5.22±0.77	8.88±0.49*	7.17±0.22*, **

Values are presented as means ± SD, ND; Normal diet, HFD; High fat diet, TE; treadmill exercise, HOMA-IR; homeostasismodel assessment-insulin resistance; AVF; abdominal visceral fat.*Denotes statistical difference from the ND-Con group.

*Denotes statistical difference from the ND-Con group.

**Denotes statistical difference from the HFD-Con group (*p* <0.05).

### The effects of TE on the cognitive function (working memory) of the HFD-fed rats

**Fig.1.**
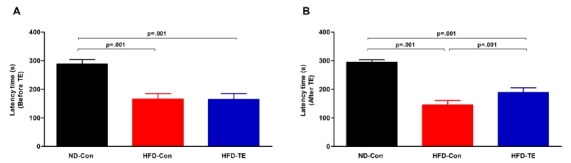
Efects of treadmill exercise on working memory in high-fat die(Ht FD)-fed rats. (A) Before treadmill exercise, (B) After treadmill exercise. The test animals performed a passive avoidance task 3 days before the end of the treadmill exercise to measure their working memory ability. The data shown for the retention latency time represent the means from 12 rats. Bonferroni’s post-hoc test was performed after analysis of variance (ANOVA). Values are presented as mean ± standard deviation (SD).

The results of the passive avoidance test that was used to evaluate cognitive function are shown in Figure 1. First, passive avoidance testing before TE revealed significant differences among the ND-Con, HFDCon, and HFD-TE groups [*F*_(2,35)_=173.363, *p*=.001]. Moreover, passive avoidance testing after TE also showed significant differences among the three groups [*F*_(2,35)_=363.775, *p*=.001].

### The effects of TE on the expression of tau protein in the cerebral cortex of the HFD-fed rats

**Fig.2.**
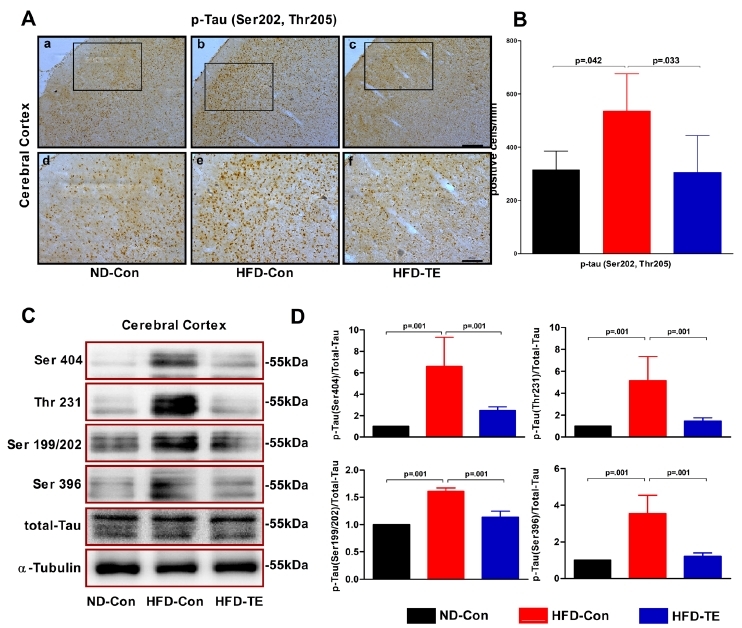
Efects of treadmill exercise on the expression of phosphorylate dtau protein in HFD-fed rats (A) Photomicrographs showing phospho-tau (Ser202/Thr205) immunoreactivity in the cerebral cortex of HFD-fed rats in each grou. p(B) Quantitative analysis of phospho-tau (Ser202, Thr205) protein in the cerebral cortex. The data shown forimmunohistochemistry represent the means from five rat brains.(C) Representative western blots ofthe Ser 404, Ser396, Thr 231, and Ser 199/202 proteins, (D) Densitometric analysis of the western blot bands, normalized to total tau. The data shown in the western blot represent the means from seven rat brains. α-Tubulin was probed as an internal control. Bonferroni’s posthoc test was performed afterA NOVA. Values are presented as mean ± SD.(Aa–c) Scale bar = 200 μm. (Ad–f) Scale bar = 100 μm.

We performed an ANOVA using the immunohistochemistry results for p-tau (Ser202, Thr205) (Figure 2-A,B). We observed significant differences among the ND-Con, HFD-Con, and HFD-TE groups in the cortex [*F*_(2,14)_=5.788, *p*=.017]. We also performed ANOVAs on the phosphorylation of the different tau phosphorylation sites (Ser404, Thr231, and Ser199/202, Ser396) (Figure 2-C,D). Significant differences were observed among the ND-Con, HFD-Con, and HFD-TE groups for Ser404 phosphorylation [*F*_(2,20)_=23.681, *p*=.001], Thr231 phosphorylation [*F*_(2,20)_=22.160, *p*=.001], Ser199/202 phosphorylation [*F*_(2,20)_=131.846, *p*=.001], and Ser396 phosphorylation [*F*_(2,20)_=40.876, *p*=.001].

### The effects of TE on the expression of insulin signaling-related proteins in the cerebral cortex of the HFD-fed rats

**Fig.3.**
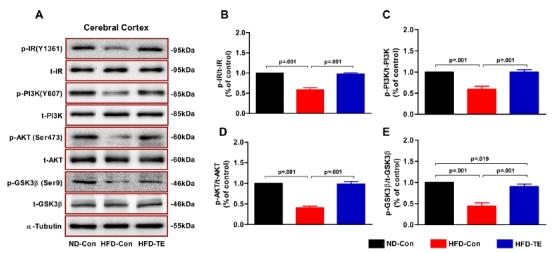
Efects of treadmill exercise on the expression of brain insulinsi gnaling-related proteins in the cerebral cortex of HFD-fed rtas. (A) The total abundances of the proteins IR, PI3K, AKT, and GSK-3β in cerebral cortexextracts were measured using western blotting and (B–E) quantitative analysis.The phosphorylation levels of IR, PI3K, AKT, and GSK-3β were normalized to total IR, PI3K, AKT, and GSK-3β.α-Tubulin was used as a control for total loading. Statistical analysis was calculatedb y one-way ANOVA followed by Bonferronis’ post-hoc test.

We performed ANOVAs to compare the phosphorylation of insulin signaling-related proteins (IR, PI3K, Akt, and GSK3β) (Figure 3). We observed statistically significant differences among the ND-Con, HFD-Con, and HFD-TE groups in terms of IR phosphorylation (p-IR/t-IR) [*F*_(2,20)_=316.150, *p*=.001], PI3K phosphorylation (p-PI3K/t-PI3K) [*F*_(2,20)_=148.290, *p*=.001], Akt phosphorylation (p-Akt/t-Akt) [*F*_(2,20)_=509.365, *p*=.001], and GSK-3β phosphorylation (p-GSK-3β/t-GSK-3β) [*F*_(2,20)_=193.496, *p*=.001].

## DISCUSSION

In the present study, we induced obesity by feeding rats a long-term (20 weeks) HFD; we then investigated the effects of TE (eight weeks) on various pathological phenomena. First, we found that TE reduced AVF and improved IR. In addition, TE restored the expression of brain insulin signaling- related proteins and alleviated the hyperphosphorylation of tau, which suggested that TE was able to prevent cognitive dysfunction. Here, we discuss these results in further detail.

The long-term administration of an HFD caused increases in BW and AVF in the experimental animals, andthese changes were accompanied by a worsening of the insulin resistance marker HOMA-IR and blood glucose metabolism (represented by the AUC calculated from the oral glucose tolerance test data), as compared with the ND-Con group. Ultimately, the long-term HFD negatively impacted energy metabolism, as compared with the ND. However, following the HFD, eight weeks of TE resulted in decreases in BW and AVF, as well as improvements in the HOMA-IR and AUC. These results were consistent with those of previous studies ^19^. Therefore, our results support the contention that obesity promotes metabolic disease, whereas aerobic exercise can reduce BW via fat loss and improve insulin resistance by improving blood glucose metabolism^20^. Our results demonstrated that diet-induced pathological energy metabolism can be prevented and ameliorated by TE.

The PI3K/Akt/GSK-3β pathway, which is a brain insulin signaling pathway downstream of IR and a tau phosphorylation pathway in AD, is thought to be involved in insulin signaling, tau hyperphosphorylation, and, by extension, the onset of AD^21^. Beginning with IR, cell survival and metabolism are controlled by the PI3K/Akt signaling pathway. GSK-3β plays an important role in the control of tau phosphorylation, and a decrease in the activity of GSK-3β following its phosphorylation by Akt kinase results in reduced tau phosphorylation. Notably, GSK-3β targets the Ser199, Thr231, Ser396, and Ser413 phosphorylation sites on tau; moreover, it is the primary tau kinase that is involved in tau overexpression^22^. The brain insulin signaling-related proteins IR, PI3K, Akt, and GSK-3β all exhibited decreased phosphorylation in the HD-Con group, as compared with the ND-Con group. However, TE reversed these changes, thus leading to an increased expression of these brain insulin-related phosphorylation [(Ser202/Thr205, Ser202, Ser306, Thr231, and Ser199/202], whereas TE reduced tau phosphorylation. These results were consistent with those of previous studies that reported a positive effect of TE on tau phosphorylation^23,24^. Takashima^22^ reported that tau hyperphosphorylation via GSK-3β was associated with insulin resistance in the central nervous system; this suggested, as implied by our study, that impaired brain insulin signaling could contribute to tau hyperphosphorylation. Hence, TE improves brain insulin signaling function, which results in a balanced phosphorylation and dephosphorylation of tau, thereby maintaining the neuronal skeleton and stabilizing the capillaries involved in the transport of neurotransmitters.

In the passive avoidance task used to evaluate memory impairments, the retention latency time until the rat entered the dark chamber was shorter in the HF-Con group than in the ND-Con group, which indicated memory impairments. Previous studies have shown that obesity increases peripheral insulin resistance and causes central insulin resistance by decreasing the levels of brain insulin; importantly, peripheral insulin resistance is associated with cognitive impairment^25,26^. In rodents, a HFD results in cognitive and memory impairments. Thus, obesity, which can affect cognitive functions either directly or indirectly, is a major cause of cognitive impairment.

In the present study, we investigated brain insulin signaling and tau expression in experimental animals that exhibited obesity induced by a long-term HFD, rather than by genetic mutations or drug treatments. The long-term HFD resulted in pathological phenomena including brain insulin dysfunction, tau hyperphosphorylation, and memory impairments. Thus, HFD-induced obepositive sity negatively impacted brain function. The most notable finding was that tau hyperphosphorylation, which is a representative pathological feature of AD, was observed in the HFD animals. Our results indicated that obesity can be a major factor in AD and other forms of dementia that involve cognitive and memory impairments. However, additional research is required to determine whether obesity is a direct cause of AD and dementia, and the indication of this potential link is an important outcome of this study.
